# Supplementary material for: Patients’ Experiences and Communication with Teledermatology versus Face-to-Face Dermatology
Source: J Clin Med. 2022 Sep 21;11(19):5528. doi: 10.3390/jcm11195528 (PMC9573490; doi:10.3390/jcm11195528)
Supplement: Supplementary file 1 [file jcm-11-05528-s001.zip › jcm-1911874-supplementary.pdf]

Table S1. Results derived from patient experiences and communication questionnaire.

	Teledermatology			Face-to-Face Dermatology					
	Min	Max	Mean	Min	Max	Mean	p-value	Effect size	Interpretation
Q1. Did the clinicians talk to you in a way that was easy to understand?	1	5	4.37	3	5	4.47	0.20	-	-
Q2. Do you have confidence in the clinicians' professional skills?	1	5	4.49	1	5	4.34	<0.01	0.11	Very small
Q3. Did you get sufficient information about your diagnosis/afflictions?	1	5	4.36	3	5	4.59	<0.01	0.16	Small
Q4. Did you perceive the treatment as adapted to your situation?	1	5	4.36	1	5	4.28	0.60	-	-
Q5. Were you involved in decisions regarding your treatment?	1	5	3.66	3	5	4.58	<0.01	0.42	Large
Q6. Did you perceive the institution's work as well organized?	1	5	3.60	1	5	2.86	<0.01	0.31	Moderate
Q7. Did you have to wait before you were admitted for services at the institution?	3	5	4.33	3	5	4.31	0.65	-	-
Q8. Overall, was the help and treatment you received at the institution satisfactory?	1	5	2.69	3	5	4.42	<0.01	0.55	Large
Q9. Overall, what benefit have you had from the care at the institution?	1	4	1.20	1	3	1.12	0.07	-	-
Q10. Do you believe that you were in any way given incorrect treatment? *	1	5	2.81	1	4	1.24	<0.01	0.53	Large
Q11. I was asked questions in an aggressive manner*	1	1	1.00	1	1	1.00	1.00	-	-
Q12. I was given answers in an aggressive manner*	1	1	1.00	1	1	1.00	1.00	-	-
Q13. I was treated with kindness	1	5	4.50	2	5	4.12	<0.01	0.26	Moderate
Q14. I was treated in a rude and hasty manner*	1	5	1.45	1	3	1.51	<0.01	0.04	Very small
Q15. The healthcare provider addressed me with a smile	2	5	3.90	1	4	2.70	<0.01	0.69	Large
Q16. The healthcare provider was able to manage the consultation	1	5	3.69	1	5	3.28	<0.01	0.28	Moderate
Q1. Did the clinicians talk to you in a way that was easy to understand?	3	5	4.97	5	5	5.00	0.014	0.11	Very small

*Negative items
